# Mitochondrial and sex steroid hormone crosstalk during aging

**DOI:** 10.1186/2046-2395-3-2

**Published:** 2014-02-05

**Authors:** Michael C Velarde

**Affiliations:** 1 Buck Institute for Research on Aging, 8001 Redwood Blvd, Novato, CA 94945, USA

**Keywords:** Mitochondrial damage, Estrogen, Progesterone, Testosterone, Longevity, Reproductive aging, Reactive oxygen species

## Abstract

Decline in circulating sex steroid hormones accompanies several age-associated pathologies which may influence human healthspan. Mitochondria play important roles in biosynthesis of sex steroid hormones, and these hormones can also regulate mitochondrial function. Understanding the cross talk between mitochondria and sex steroid hormones may provide insights into the pathologies associated with aging. The aim of this review is to summarize the current knowledge regarding the interplay between mitochondria and sex steroid hormones during the aging process. The review describes the effect of mitochondria on sex steroid hormone production in the gonads, and then enumerates the contribution of sex steroid hormones on mitochondrial function in hormone responsive cells. Decline in sex steroid hormones and accumulation of mitochondrial damage may create a positive feedback loop that contributes to the progressive degeneration in tissue function during aging. The review further speculates whether regulation between mitochondrial function and sex steroid hormone action can potentially influence healthspan.

## Review

### Introduction

Sex steroid hormones play important roles in maintaining normal reproductive and non-reproductive functions. Because aging is accompanied by decreasing levels of circulating sex steroid hormones (that is, estrogen, progesterone, and testosterone) [1,2], many researchers and clinicians had previously hypothesized that the decline in these hormones promotes tissue degeneration and age-related pathologies. As a result, hormone replacement therapies have already been used to alleviate age-related symptoms in men and women. However, the efficacy of these treatments remains debatable. Hence, it is important to evaluate the contribution of sex steroid hormones on healthspan.

Mitochondria regulate sex steroid hormone biosynthesis, and sex steroid hormones are also implicated in controlling mitochondrial function. This co-regulation between mitochondria and sex steroid hormone signaling is important for maintaining proper physiology. Disrupting the crosstalk between mitochondrial function and sex steroid hormone action may alter cellular responses and accelerate aging-associated phenotypes. Understanding the relationship between mitochondria and sex steroid hormones may provide insights into the efficacy of using hormone replacement therapies to treat age-related diseases. This review summarizes the current knowledge regarding the crosstalk between mitochondria and sex steroid hormones during aging, and examines whether the interplay between mitochondrial function and sex steroid hormone action can potentially influence healthspan.

## Sex steroid hormones and aging

### Sex steroid hormone production declines during reproductive aging

Reproductive aging is defined as the gradual decline in fertility during the aging process. It is accompanied by decreasing levels of circulating sex steroid hormones estrogen, progesterone, and testosterone [1,2]. In women, the end of reproductive aging occurs when ovarian follicles drop below a threshold (approximately < 1,000 follicles), resulting in cessation of the menstrual cycle which is termed menopause [3]. The stock of non-renewing ovarian follicle reserves begins to decline from birth as a result of continuous maturation, ovulation, and atresia [4]. Ovarian follicles eventually become exhausted by the age of 51, which corresponds to the median age of menopause in the general population [5]. Because granulosa cells and theca cells of the ovarian follicles are the major sources of sex steroid biosynthesis in women, reproductive aging eventually leads to reduced estrogen and progesterone production [6], although the drop in progesterone levels surpasses the drop in estrogen levels during the menopausal transition [7].

In men, reproductive aging is accompanied by a decline in the ability of the testes to produce testosterone [8]. Leydig cells of the testes provide a significant source of testosterone in males. Reduced number of Leydig cells and decreased ability to respond to intracellular signaling during aging may contribute to low testosterone levels in aging men [9-11]. Testosterone production gradually decreases by approximately 1%–3% per year, beginning around the age of 35–40 years [12], and can eventually result in hypogonadism (<325 ng/dL of total serum testosterone) in older men. Partial androgen deficiency (sometimes referred as andropause) occurs in ~20% of men by age 60 and ~50% of men by age 80 [13].

### Sex steroid hormone replacement may extend healthspan but not lifespan

The decline in sex steroid hormones with age is thought to reduce healthspan. In women, menopause correlates with several age-related diseases, such as hot flashes, bone loss, weight gain, vaginal atrophy, night sweats, sleep problems, mood changes, and skin atrophy [14,15]. Estrogen replacement alleviates some of these menopausal symptoms, resulting in lowered frequency and severity of hot flashes, decreased bone loss and vertebral fractures, and increased bone mass [16]. Estrogen can also provide many beneficial effects to skin physiology and protects against skin aging [17]. In female mice, loss of sex steroid hormones by ovariectomy also presents numerous age-related diseases similar to those found in humans, such as accelerated bone loss, decreased uterine weight, increased fat mass, and impaired wound healing, which can all be alleviated by estrogen replacement [18-20].

Because estrogen increases the risk of endometrial cancers to women, progesterone supplements were added to estrogen replacement therapies to inhibit the proliferative effects of estrogen in the uterus. Estrogen plus progesterone replacement therapy provides many beneficial effects similar to those of estrogen alone treatment, but it also seems to increase the risk for other types of diseases, such as breast cancer [16]. Findings from the Women’s Health Initiative show a high risk-to-benefit ratio of using hormone replacement therapies. However, criticisms of the regimens used suggest that health status or age at initiation of treatment may contribute to severity of the side effects associated with hormone replacement therapy [21,22]. Analysis of the current Kronos Early Estrogen Prevention Study (KEEPS) [23] and the Early versus Late Intervention Trial with Estradiol (ELITE) will hopefully address whether hormone replacement therapy is beneficial within a critical window of time [24,25].

In men, hypogonadism at old age is correlated with age-related symptoms, such as impotence, decreased virility, decreased libido, and limited physical vigor [26]. However, it remains debatable whether these age-related phenotypes are mainly due to the decline in testosterone levels. In fact, while testosterone replacement can alleviate some of the aging characteristics associated with partial androgen deficiency [27-31], testosterone supplementation in aged men does not always seem to improve physical function [32]. Moreover, increased skeletal muscle mass due to testosterone does not seem to be specific to aged men [33]. In male mice, testosterone can increase skeletal muscle regeneration [34], but it may also accelerate aging phenotypes in other tissues, such as heart and kidneys [35]. Hence, the overall impact of testosterone on healthspan needs further investigation. The current Testosterone Trial will hopefully address the benefit and risk of testosterone replacement in hypogonadal men above the age of 65.

While sex steroid hormones may potentially extend healthspan, the effect of sex steroid hormones on lifespan is less clear. For example, while increasing age at menopause in women is associated with an overall increased lifespan [36], menopausal age does not seem to predict lifespan in non-human primates, as chimpanzees rarely reach menopause whether in captivity or in the wild [37,38]. Moreover, the length of postmenopausal years (that is, period of reduced sex steroid hormones) does not seem to be proportionally correlated with age at death [39]. In males, castration in rats and dogs increases lifespan [40,41]. However, while some studies show that castration in humans (that is, eunuchs) extends lifespan [42,43], another report mentions no change in lifespan [44]. Nevertheless, several studies have linked reduced sex steroid hormones to many age-related diseases. Hence, while loss of sex steroid hormones may not significantly influence longevity and lifespan, reduced sex steroid hormones can potentially shorten overall healthspan.

## Mitochondria and sex steroid hormone biosynthesis

### Sex steroid hormone biogenesis

Mitochondria play an essential role during the initial steps of sex steroid hormone biosynthesis, particularly by producing the sex steroid hormone precursor pregnenolone. Import of cholesterol from the outer to the inner mitochondrial membrane is a rate limiting step during the initial biosynthesis of sex steroid hormones [45]. Cholesterol transport involves interaction between the steroidogenic acute regulatory protein (StAR) and a multi-component molecular complex, which is composed of an 18 kDa translocator protein (TSPO), the voltage dependent anion channel, TSPO-associated protein 7, and protein kinase A subunit 1a [46]. Once imported, cholesterol is converted to pregnenolone by the cytochrome P450 side-chain cleavage (P450scc; or CYP11A1) enzyme, located on the inner membrane of the mitochondria, involving the oxidation of nicotinamide adenine dinucleotide phosphate (NADPH) [47]. Pregnenolone can then be exported out of the mitochondria and converted by specific microsomal P450 enzymes into the different sex steroid hormones [48].

In males, testosterone biogenesis mostly occurs in Leydig cells through the sequential conversion of pregnenolone to 17α-hydroxypregnenolone, dehydroepiandrosterone (DHEA), androstenedione, and testosterone [47]. In females, however, estrogen and progesterone biosynthesis occurs through the crosstalk between the granulosa and theca cells of the ovarian follicle. Pregnenolone in granulosa cells can diffuse to theca cells and be converted into androstenedione, which then re-diffuses back to granulosa cells and is eventually processed to estrogen. In some instances, androstenedione produced by the theca cells can also be immediately converted to testosterone in these cells. During the luteal phase of the menstrual cycle, progesterone is produced in luteinized granulosa cells by direct conversion from pregnenolone.

### Mitochondrial damage may promote menopause and partial androgen deficiency

Mitochondria can produce reactive oxygen species (ROS) at multiple sites of the electron transport chain [49]. During mitochondrial respiration, the electron transport chain generates a flux of electrons, capable of establishing a proton gradient within the mitochondria. While this proton gradient is necessary for ATP production, electrons produced during oxidative phosphorylation need to be completely neutralized to water upon reaction with oxygen at complex IV (that is, cytochrome c oxidase). However, partial reduction of oxygen can occur upstream of complex IV, resulting in production of superoxides. These superoxides are normally eliminated by antioxidants (that is, superoxide dismutases, SOD) in the mitochondria. However, in certain conditions, some superoxides are thought to escape the mitochondrial antioxidant system and cause damage to mitochondrial proteins and DNA. Damaged mitochondria become less efficient in transferring electrons across the respiratory chain, rendering more electrons to be converted to superoxides. This positive feedback loop of mitochondrial superoxide (ROS) production and mitochondrial damage was proposed to cause aging, a theory known as the free radical theory of aging [50,51]. Consistent with this theory, damage to mitochondrial DNA, lipids, and proteins increases with age, and is accompanied by decreased mitochondrial membrane potential and impaired mitochondrial function [52,53].

In women, drop in sex steroid hormones during menopause is mainly attributed to loss of ovarian follicles. Continuous ovulation and follicular atresia, plus inability of follicles to naturally regenerate, eventually lead to reduced sex steroid hormone production. Aside from this progressive decline in follicle numbers, it is suggested that excessive oxidative damage in the ovaries, due to a decrease in the levels of antioxidants, may be a potential contributing factor for reproductive aging [54]. While it is yet to be shown whether mitochondrial ROS generation promotes mitochondrial damage in ovarian follicles, increased number of damaged mitochondria have been observed in ovarian follicles of aged women [55,56] and the proportion of granulosa cells with ruptured mitochondrial membranes significantly increase with age [55]. While human studies only reveal correlations between mitochondrial damage and loss of ovarian follicles, mouse studies show that mitochondrial damage can contribute to reproductive aging. Mitochondrial DNA damage in a mouse model with defective mitochondrial DNA polymerase can reduce female fertility [57]. Mitochondrial dysfunction in a mouse model with high mitochondrial ROS generation also results in infertility, defective folliculogenesis, and impaired ovulation [58]. Hence, mitochondrial damage may potentially accelerate the decline in ovarian follicles and partly contribute to reproductive aging in females.

In men, drop of testosterone levels during aging is associated with decreasing supply of mitochondrial steroid precursors by Leydig cells [9,10]. In male rats, testosterone levels have also been shown to decline with age [59]. This age-related decline in testosterone steroidogenesis is consistent with decreased mitochondrial expression of StAR and CYP11A1 in Leydig cells of old versus young rats [60]. While the cause of this decline in StAR and CYP11A1 expression is still unclear, it has been proposed that ROS may contribute to this effect. Increased mitochondrial ROS was observed in Leydig cells of old versus young rats [61]. Transient elevation of ROS production after luteinizing hormone treatment caused more DNA damage in aged versus young Leydig cells [62]. Moreover, ROS (that is, H_2_O_2_) can inhibit testosterone biosynthesis in the mouse Leydig cell line MA-10 and a primary rat Leydig cell line [63,64]. But because the concentration used is relatively high (100 to 250 μM), the physiological relevance of this ROS-inhibition to steroidogenesis remains questionable. Nevertheless, while the biological impact of mitochondrial ROS on steroidogenesis needs further validation, the role of mitochondria in producing steroid precursors suggests that properly functioning mitochondria are important to maintain sex steroid hormone levels. Hence, there is a need to critically evaluate the contribution of mitochondrial damage on sex steroid hormone production during aging.

## Sex steroid hormones and mitochondrial function

### Sex steroid hormone nuclear receptors regulate mitochondrial gene expression

**Figure 1:**
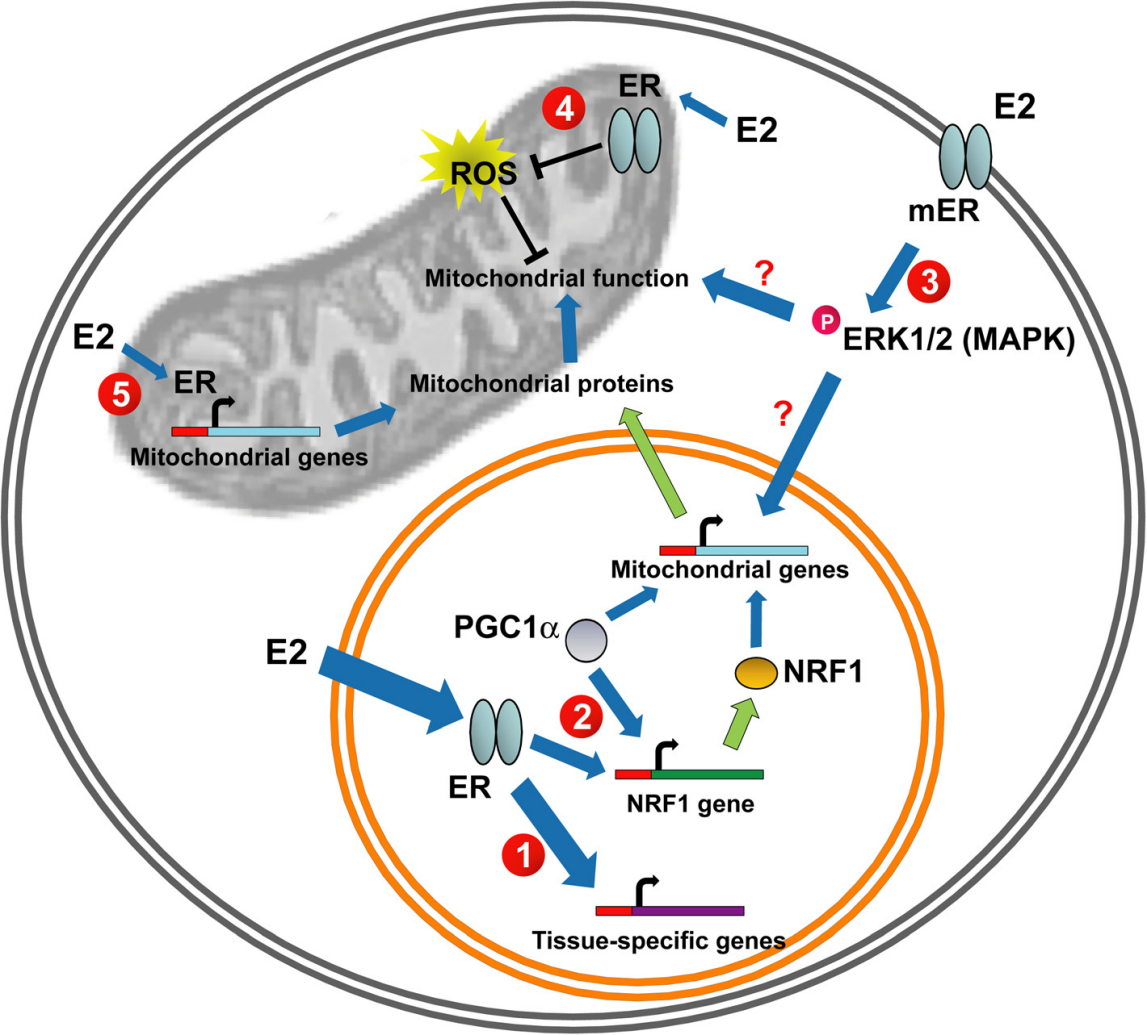
**Molecular targets of estrogen in regulating mitochondrial function.** 1. Estrogen binds to nuclear estrogen receptors (ERα and ERβ) to directly regulate tissue-specific expression of genes necessary for cellular function. 2. Estrogen binds to nuclear ERs and regulates expression of mitochondrial genes by promoting gene expression of transcription factor NRF1 and enhancing transcriptional activity of PGC1α. 3. Estrogen binds to membrane ERs and activates signaling cascades that protect mitochondria from oxidative damage. 4. Estrogen binds to mitochondrial ERs and limits ROS generation in the mitochondria. 5. Estrogen binds to mitochondrial ERs and regulates transcription of mitochondrial-encoded mitochondrial genes.

While mitochondria mediate sex steroid hormone production, sex steroid hormones can also regulate mitochondrial function. Sex steroid hormones estrogen, progesterone, and testosterone classically function by binding to their nuclear receptors: estrogen receptors (ERα and ERβ1-6), progesterone receptors (PR-A and PR-B), and androgen receptors (AR1 and AR2), respectively. These receptors along with specific nuclear receptor co-regulators can directly or indirectly bind to nuclear DNA regulatory elements and influence gene expression [65]. While the direct contributions of testosterone and progesterone on mitochondrial function are less studied, the effect of estrogen on mitochondrial function and biosynthesis is more evident. Estrogen, through its receptor, can directly modulate expression of genes important for mitochondrial function (Figure 1). Estrogen can directly up-regulate transcription of nuclear respiratory factor-1 (NRF1), a key transcription factor necessary for regulating expression of most of the mitochondrial respiratory chain complex proteins [66]. NRF1 promoter contains putative estrogen receptor response elements capable of binding both ERs [66,67]. ERα can also interact with peroxisome proliferator-activated receptor gamma coactivator 1 (PGC-1), another important transcription factor that promotes transcription of NRFs and other mitochondrial proteins [68]. Regulation of NRFs and PGC-1 by estrogen is thought to play an important role in modulating overall mitochondrial biogenesis and function [69].

### Sex steroid hormone membrane receptors protect against mitochondrial oxidative damage

Aside from the function of sex steroid hormones in the nucleus, sex steroid hormones can also have rapid, nongenomic actions by activating plasma membrane-associated receptors, leading to intracellular protein kinase-mediated phosphorylation signaling cascades [70]. While studies regarding the contribution of plasma membrane-associated receptors on mitochondrial function are very limited, some reports suggest a role for this signaling in mitochondria. Estrogen, by acting on plasma membrane-associated ER, is thought to trigger a phosphorylation cascade and limit mitochondrial oxidative damage (Figure 1). One of the early studies suggesting this phenomenon was when a BSA-conjugated form of estrogen, which is membrane-impermeable, was able to undergo rapid internalization and translocation into mitochondria but not the nucleus [71,72]. Estrogen, acting through intracellular phosphorylation cascades, may protect mitochondria from oxidative damage. One study shows that estrogen can activate the MAP kinase and NF-kB pathways to reduce cellular levels of hydrogen peroxides by stimulating the nuclear transcription of mitochondrial antioxidant enzyme *Sod2*[73]. However, it remains to be confirmed whether this estrogen-mediated *Sod2* mRNA regulation is necessary to protect against mitochondrial oxidative damage. Another study also shows that estrogen can activate ERK, permit its translocation to the mitochondria, and enhance cytochrome c oxidase complex IV activity [74]. Whether these estrogen actions are due to plasma membrane-associated receptors and whether they play a significant role in protecting mitochondria against oxidative damage needs further investigation.

### Mitochondria-associated sex steroid hormone receptors protect against mitochondrial oxidative damage

Sex steroid hormone receptors, particularly ERs, have been observed to localize in mitochondria and contribute to mitochondrial function (Figure 1). Despite the controversy regarding mitochondrial localization of ERs [75,76], accumulating evidence suggest that both ERα and ERβ are indeed present in the mitochondria, depending on cell type [77-80]. ERβ seems to be the ER that is more frequently present in mitochondria of most cell types [81-85]. Knockdown of the ERβ1 isoform, which predominantly localizes to mitochondria, eliminates estrogen-dependent protection against peroxide-induced mitochondrial membrane depolarization [86]. Using a targeting vector containing mitochondrial or nuclear localization sequences, ER can be efficiently targeted to mitochondria or nucleus [84]. Expression of a mitochondrial-targeted, but not a nuclear-targeted ER, confers estrogen-dependent inhibition of UV-induced mitochondrial depolarization in a breast cancer cell line MCF-7 by enhancing mitochondrial SOD2 protein activity, independent of its transcriptional regulation [84]. However, the mechanism of ER in regulating SOD2 protein activity remains unclear and needs further confirmation.

Sex steroid hormone receptors in the mitochondria can regulate transcription of mitochondrial encoded genes [87]. Mitochondrial DNA contains hormone response elements, which allow binding of steroid hormone receptors. Indeed, ER in the mitochondria can bind to estrogen response elements (ERE) located in mitochondrial DNA (Figure 1) [88]. This estrogen-mediated ER binding to mitochondrial DNA is thought to increase expression of mitochondrial-encoded mitochondrial genes associated with the electron transport chain [89]. Aside from regulating transcription of mitochondrial encoded genes, sex steroid hormone receptors have been suggested to bind to mitochondrial proteins. For example, ERβ has been shown to co-immunoprecipitate with the mitochondrial protein ATP synthase [90]. However, whether this interaction is functional remains to be addressed.

### Hormone replacements improve mitochondrial function in healthy cells

Estrogen can inhibit mitochondrial ROS generation in primary cells, such as endothelial cells, cardiomyocytes, and epithelial lens cells [86,91-93]. Animal experiments also demonstrate that estrogen can reduce mitochondrial ROS production and enhance mitochondrial respiration in normal brains of male and female rats [94,95]. Estrogen seems to limit mitochondrial ROS production in cell types, which are predominantly expressing ERβ, but a few studies have also supported a protective role of ERα [96].

While estrogen replacement seems to provide overall beneficial effects on mitochondrial function, timing of treatment and type of cell may be important for this phenomenon. Estrogen can decrease mitochondrial ROS production in non-cancer primary cells, but it can also increase ROS production in damaged estrogen-responsive cancer cells [97-99]. This suggests that while estrogen can protect normal cells from oxidative stress, it exacerbates oxidative stress in damaged cells, that is, cancer cells. While it remains unclear what mechanisms regulate this contradictory effect of estrogen on mitochondrial ROS production, the contribution of damaged versus healthy cell on estrogen action is consistent with the critical window and healthy cell hypothesis of estrogen replacement therapy [22,100]. This hypothesis proposes that estrogen replacement is only beneficial if performed at the appropriate time of a woman’s life, before she accumulates a certain threshold of cellular damage. Indeed, estrogen replacement therapies seem to be more beneficial in younger versus older women [25]. This is one of the rationales for the current KEEPS and ELITE studies on estrogen replacement therapies [23]. Results from these studies will hopefully address some of these questions.

The effect of testosterone replacement on mitochondrial function is less well understood. Low levels of testosterone in males seem to be associated with reduced expression of mitochondrial respiratory genes and activity [101]. Orchiectomy in young male mice decreases expression of genes associated with energy metabolism, oxidative phosphorylation, and ubiquinone pathways [102]. AR overexpression in myocytes increases mitochondrial enzyme activities and oxygen consumption [103,104]. Testosterone therapy potentiates the effect of low-intensity physical training in old male mice by increasing mitochondrial biogenesis, improving mitochondrial quality, and increasing spontaneous physical activity, respiration, muscle mass, and grip strength [105]. However, testosterone has also been shown to reduce mitochondrial function [106]. Whether or not testosterone play a major role in mitochondrial function needs further investigation.

## The positive feedback loop of mitochondrial damage

**Figure 2:**
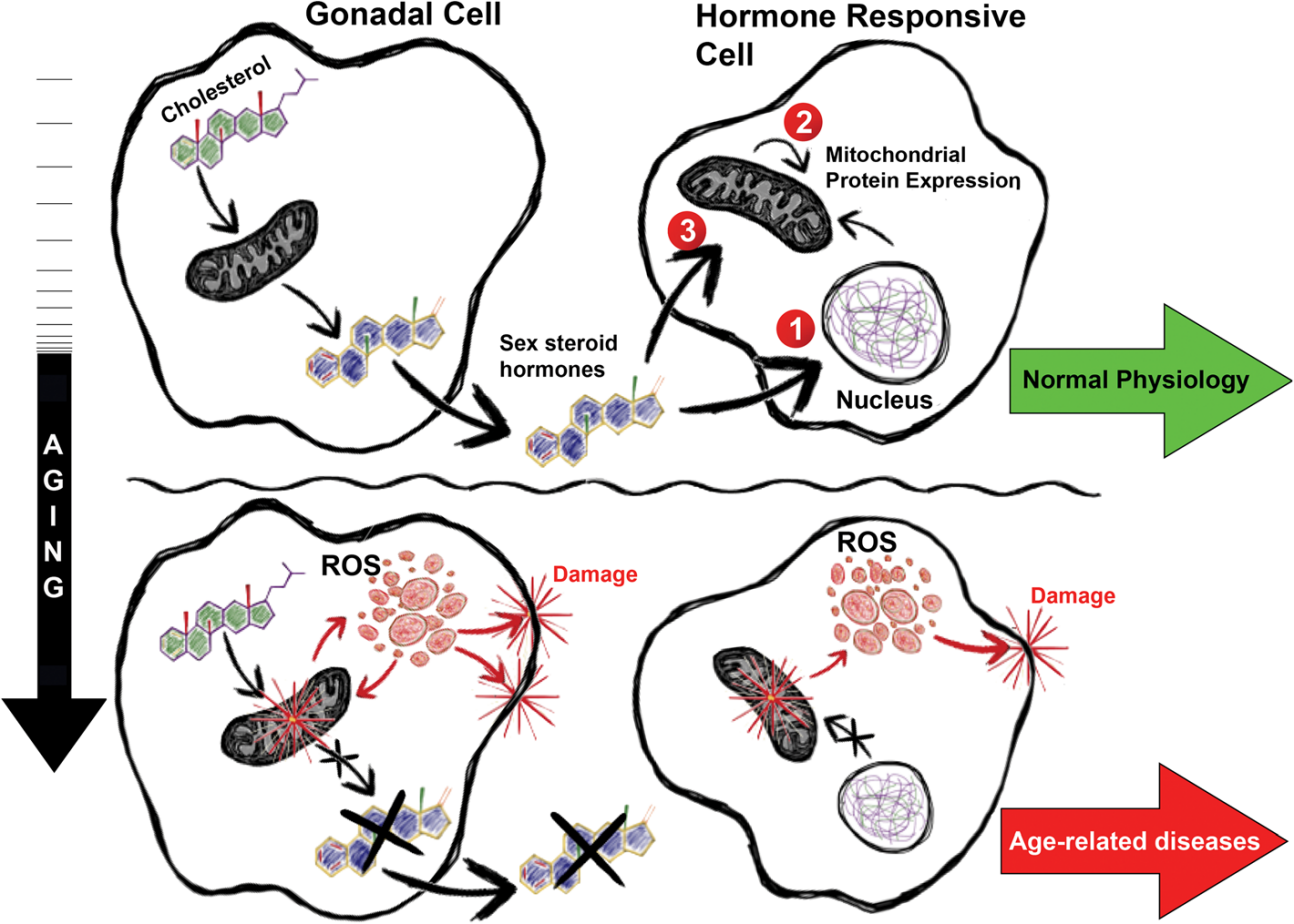
**Mitochondria and sex steroid hormones during aging.** Mitochondria produce sex steroid hormones in the gonads through initial conversion of cholesterol to pregnenolone. Sex steroid hormones improve and/or maintain mitochondrial function in hormone responsive cells by regulating: 1. gene expression of nuclear-encoded mitochondrial proteins, 2. gene expression of mitochondrial-encoded mitochondrial proteins, and/or 3. activity of mitochondrial proteins. Increased oxidative stress during aging damages gonadal cells and/or impair steroidogenesis. Decline in sex steroid hormone biosynthesis during aging compromises mitochondrial function in hormone responsive tissues and contribute to age-related pathologies.

Mitochondria play important roles in the biosynthesis of sex steroid hormones by housing important enzymes for steroidogenesis and by maintaining cells that produce these hormones (Figure 2). Conversely, sex steroid hormones help to maintain mitochondrial function and limit aging phenotypes. According to the free radical theory of aging, mitochondrial ROS produced during cellular respiration can result in mitochondrial damage, which then initiates a positive feedback loop of increased mitochondrial ROS generation and mitochondrial damage with age. Accumulation of damaged mitochondria can compromise steroidogenesis by damaging gonadal cells, resulting in decreased sex steroid hormone levels. Decline in sex steroid hormones exacerbates mitochondrial dysfunction and further promotes mitochondrial damage. This cycle of mitochondrial damage and decline in sex steroid hormone levels can potentially contribute to the progressive degeneration in tissue function during aging.

While the free radical theory provides a potential model for the cause of aging, several studies have questioned whether accumulation of mitochondrial oxidative damage actually drives the aging process [107,108]. Some studies support the theory [109,110], but others suggest that increased mitochondrial ROS production does not decrease lifespan [111], but can even potentially extend lifespan [112]. It is possible that depending on the amount and context, mitochondrial ROS production can provide beneficial or detrimental effects. Excessive mitochondrial ROS may result in cellular damage and aging, but low mitochondrial ROS production may provide a hormetic effect that extends lifespan [113]. Further research needs to be performed to confirm the validity of the theory.

It is also important to note, that while this review focused on the effects of sex steroid hormones on mitochondrial function, sex steroid hormones can also regulate other cell function independent of mitochondria (Figure 1). Moreover, while estrogen can directly and indirectly regulate mitochondrial biogenesis and function, it remains to be confirmed whether testosterone can significantly contribute to mitochondrial function.

## Conclusions

Current studies show that mitochondria are important for the initial step of steroidogenesis, and sex steroid hormones (that is, estrogens) are capable of regulating mitochondrial biogenesis and function. Dysregulation of mitochondrial function and sex steroid hormone action may compromise cellular integrity and lead to progressive decline in tissue function. While the positive feedback loop of mitochondrial damage has been suggested to partly contribute to aging and age-related phenotypes, several questions still need to be addressed to validate this theory. Nevertheless, research on this topic may potentially provide insights into extending healthspan in humans.

## Abbreviations

KEEPS: Kronos Early Estrogen Prevention Study; ELITE: Early versus Late Intervention Trial with Estradiol; StAR: Steroidogenic acute regulatory protein; TSPO: Translocator protein; P450scc: Cytochrome P450 side-chain cleavage; CYP11A1: Cytochrome P450, family 11, subfamily A, polypeptide 1; NADPH: Nicotinamide adenine dinucleotide phosphate; DHEA: Dehydroepiandrosterone; ROS: Reactive oxygen species; SOD: Superoxide dismutase; ER: Estrogen receptor; PR: Progesterone receptor; AR: Androgen receptor; NRF1: Nuclear respiratory factor-1; PGC-1: Peroxisome proliferator-activated receptor gamma coactivator 1; ERE: Estrogen response elements.

## Competing interests

The author declares that he has no competing interests.

## Authors’ contributions

MV drafted all the content of the manuscript.
